# Characterizing healthy samples for studies of human cognitive aging

**DOI:** 10.3389/fnagi.2012.00023

**Published:** 2012-09-12

**Authors:** David S. Geldmacher, Bonnie E. Levin, Clinton B. Wright

**Affiliations:** ^1^Evelyn F. McKnight Brain Institute, Department of Neurology, University of Alabama-BirminghamBirmingham, AL, USA; ^2^Evelyn F. McKnight Brain Institute, Department of Neurology, University of Miami Miller School of MedicineMiami, FL, USA

**Keywords:** cognitive aging, research methods, screening instruments, subject selection, sample heterogeneity

## Abstract

Characterizing the cognitive declines associated with aging, and differentiating them from the effects of disease in older adults, are important goals for human neuroscience researchers. This is also an issue of public health urgency in countries with rapidly aging populations. Progress toward understanding cognitive aging is complicated by numerous factors. Researchers interested in cognitive changes in healthy older adults need to consider these complexities when they design and interpret studies. This paper addresses important factors in study design, patient demographics, co-morbid and incipient medical conditions, and assessment instruments that will allow researchers to optimize the characterization of healthy participants and produce meaningful and generalizable research outcomes from studies of cognitive aging. Application of knowledge from well-designed studies should be useful in clinical settings to facilitate the earliest possible recognition of disease and guide appropriate interventions to best meet the needs of the affected individual and public health priorities.

## Introduction

The definitional boundaries of cognitive aging remain controversial and are likely to elude consensus for the foreseeable future. The ambiguities stem from the wide range of goals and scientific questions arising from different lines of inquiry. For example, epidemiological studies examining the effects of disease typically employ brief health histories and screening examinations applicable to large cohorts of people, whereas experimental studies use smaller samples and focus on a limited number of cognitive domains, In contrast, the clinician working in a medical setting evaluates individuals, each of whom present with a unique chronologic, cultural, psychosocial, educational, genetic, and medical history and a decision has to be made whether the symptoms or signs the patient expresses are “normal” (i.e., do not require evaluation and intervention) or abnormal.

An important goal for research in cognitive aging is to better characterize healthy aging to assist clinical decision-making about cognitive changes in older patients. The McKnight Brain Research Foundation therefore commissioned a working group to address challenges and opportunities in cognitive aging research and provide practical information to investigators that can improve the pace, quality, and clinical utility of research in the field. This paper is not intended to be comprehensive or a systematic review of all the factors that might affect cognitive aging. Readers interested in more exhaustive treatments of this broad, complex, and well-researched field may find the volumes edited by Hofer and Alwin (2008) and Craik and Salthouse (2008) useful. Rather, we intend to provide a brief guide to factors that should be considered as a researcher classifies an older human research participant as “healthy” or not. Additionally we provide a brief review of clinically oriented instruments frequently used to characterize the cognitive state of older research participants.

## Considering cross-sectional vs. longitudinal methods

The major temporal approaches to understanding cognitive aging are cross sectional and longitudinal designs. Cross sectional approaches can be conducted on large samples across many ages efficiently and at lower cost, but have limitations. The most important limitation from an epidemiologic perspective is that causal relationships between an exposure and an outcome cannot be determined. In addition, such studies are vulnerable to cohort effects whereby different age groups have varying life experiences (e.g., early life nutrition or educational methods). As one example, Laursen (1997) found that individuals of a given age performed better on cognitive tests than people who took the same tests when they were the same age, only a decade earlier. Cross sectional studies therefore cannot clearly define aging alone as the source of group differences, but merely identify associations between age and performance. Cross sectional methods also account poorly for the effects of variability in individual performance unrelated to aging. The complexity of this issue is illustrated by results from, the Cambridge Project for Later Life, in which 28% of participants showed improved cognitive performance over a mean 28-month follow-up period (Brayne et al., 1992).

Longitudinal studies are better at clarifying the role of aging as the source of individual differences over time, but longitudinal designs are dependent on participants' ability to return for follow-up evaluation. This can be particularly challenging in elderly samples because individuals with advanced age, lower education and SES, in worse physical health, and undergoing stress, as well as those who are more likely to have depression tend to drop out of studies (Nevid et al., 1996; Burg et al., 1997; Laursen, 1997; Mihelic and Crimmins, 1997; Schmand et al., 1997; Desmond et al., 1998; Hoeymans et al., 1998; Levin et al., 2000). Therefore, longitudinal studies are susceptible to bias associated with self-censorship. Furthermore, if participants who do not return for follow up evaluation differ from those who do, an attrition bias will confound the research. This is especially problematic for studies examining more subtle forms of cognitive impairment. An attrition bias will not only limit the study's generalizability, but can exclude individuals intended to be the major study focus (Levin et al., 2000). Therefore, adjustments for the effects of differential attrition should be included in longitudinal studies to avoid underestimates of the true rate of decline (Park et al., 2003).

Another challenge faced by researchers using either method is subclinical disease. Conceptual approaches for major age-related diseases that affect cognition, including Alzheimer's disease, Parkinson's disease, and vascular cognitive impairment (VCI) now explicitly recognize pre-symptomatic stages, during which the disease causes meaningful damage to the brain without bringing the individual to medical attention (Gorelick et al., 2011). As a result, research participants classified as “normal” may in fact be experiencing subtle changes in higher-order cognitive functions, or employing compensatory mechanisms that could influence research conclusions about what is normal. The nature of this experimental challenge is exemplified by studies showing anatomic and functional imaging abnormalities among people with a known genetic risk factor for Alzheimer's disease but who exhibit normal cognitive performance (Reiman et al., 1996; Alexander et al., 2012). Similarly, cardiovascular or stroke risk factors may lead to damage that results in significant declines in cognitive performance that may remain undetected (Llewellyn et al., 2008). For example, participants with subclinical white matter lesions, subclinical infarcts, or microbleeds may have subtle changes in cognitive performance in particular domains (e.g., executive function) and be within the normative range for others.

Longitudinal analysis can also be confounded by “right censoring,” whereby participants who are normal at the last observation are considered to be unaffected by the condition under study. This phenomenon is illustrated by the MoVIEs project, in which observation for dementia incidence in 1298 participants was ended after ten years. By the end of the observation period, dementia had developed in 199 of the participants; for the other 1099 participants, death, drop-out, or completion of the study was the end-point for observation and they were classified as non-demented (Ganguli et al., 2000). Given annual dementia incidence rates of 8–40% among the non-demented oldest old (Peltz et al., 2011), and a consensus that Alzheimer's disease pathology may be present for many years before the first symptoms emerge (Albert et al., 2011), the assumption that the 1099 “survivors” in the MoVIES cohort were truly disease-free may be inaccurate. Although not practical for most researchers, future analyses conducted on large longitudinal data sets (like MoVIES or the Framingham cohort) should include analytic methods account for expected incident dementia rates.

## Heterogeneity of samples

In attempting to characterize healthy aging samples, researchers face a myriad of complicating factors. Age itself alters the frequency and rate of cognitive change, with the oldest old showing both a more impaired baseline and a more rapid decline on most neuropsychological measures (Park et al., 2003). Unfortunately, cognitive abilities have been the least well studied in the oldest age groups and there is a striking lack of normative standards to evaluate the very old. As a result, investigators studying participants older than 80 years will turn to norms developed for younger cohorts. Mayo's Older American Normative Studies (MOANS; Lucas et al., 2005) and Whittle and colleagues (2007) study examining the oldest old (90+ years) provide valuable normative data on select subtests. Given census data indicating that the population of individuals aged 85 years and older is the fastest growing age group, there will continue to be a pressing need to develop age-appropriate and age-normed tests. In the interim, investigators carrying out research on this age group should incorporate measures that have the necessary age corrections or make sure the study design includes an age matched control group (Levin et al., 2000; Manly and Echemendia, 2007; Levin, 2009).

### Culture and language

Many studies attempt to limit the effects of culture and language on their outcomes. While there is general agreement that no tests are “culture-free,” measures have been developed to minimize cultural bias. There are two types of culture fair tests. The first employs items that are assumed to be familiar to all individuals from a variety of ethnic backgrounds. The second type eliminates the need for expressive language, i.e., the test utilizes geometric or spatial stimuli and can be administered without verbal demands through the use of gestures or demonstration. However, cultural factors are much more pervasive than language alone. Culture and educational achievement significantly influence performance even on presumably non-verbal neuropsychological tests (Rosselli and Ardila, 2003). Performance on culture fair tests is also affected by enriched or impoverished environments and there is research showing even larger group differences with non-verbal tests compared to verbal tests (Geisinger, 2003). Accurate cultural adaptation of cognitive tests is extremely labor intensive and may involve alteration of test instructions, revision of both verbal and non-verbal test content, and changes in item order (Malda et al., 2008).

### Race and ethnicity

There is general agreement that most cognitive measures used in the US were standardized on Caucasian samples and are therefore biased, rendering them not appropriate in either clinical or research settings that focus on linguistically and culturally diverse populations. US census projections show that immigration will be the driving force behind future population growth, a finding that underscores the need to develop measures that are sensitive to growing racial and ethnic diversity. Furthermore, understanding cognitive aging in this context is complicated by research showing that there are a host of social/environmental factors that also influence test performance, such as the duration of residence and experiences in the US, the country and age where an individual learned English, their primary language, and reading ability (Harris et al., 2003; Manly, 2005). Altering test score cut-points for defining normal performance among minority populations is not always useful (Parker and Philp, 2004). For example, Boone and colleagues (2007) used a data set derived from Caucasians, African Americans, Hispanics, and Asians referred to an outpatient neuropsychology clinic in Los Angeles to examine associations between ethnicity and performance on common neuropsychological measures. Although the groups were similar in the nature and severity of their presenting illnesses, and the test scores were adjusted for age and education, significant ethnic group differences were noted on one-third of neuropsychological measures, which understandably included naming, but also included tests with less linguistic loading like digit span, visual construction, and non-verbal processing speed. There is no consensus on how to best address the influence of cultural heterogeneity on assessment of cognition and there are no empirically based guidelines (Manly and Echemendia, 2007). However, measures of literacy are valuable indicators of quality of education and can help account for race/ethnic and cultural differences in studies of cognitive aging. For example, in a Northern Manhattan study sample, African Americans elders performed worse than whites across a number of neuropsychological tests when matched for years of education, but the effect was much smaller, and in most cases no longer significant, after adjusting for reading ability (Manly et al., 2002).

### Life achievement

Variability in cognitive performance within age cohorts, associated with factors such as, education, culture, and race/ethic background, has led to the concept of brain and cognitive reserves, theoretical constructs representing, respectively, the anatomical and functional factors that protect the brain from aging and pathology (Stern, 2002, 2009). In the case of cognitive reserve, a growing body of data suggests that educational and occupational attainments, as well as a stimulating lifestyle, provide resistance against the effects of aging and diverse pathologies, such as vascular damage and Alzheimer disease (Stern et al., 1994). Indeed, both higher education and occupational attainment have been associated with slower cognitive decline and a lower risk of dementia in the elderly (White et al., 1994; Evans et al., 1997; Valenzuela and Sachdev, 2006). An active lifestyle—one that includes intellectual stimulation, increased physical activity and participation in social activities—has been associated with better neuropsychological test performance in later years and a lower risk of dementia (Scarmeas et al., 2001; Verghese et al., 2003). Functional neuroimaging studies have confirmed the hypothesis that such protective factors may achieve their effect by creating more efficient cognitive networks and promoting neural compensation (Steffener et al., 2011). By accounting for these factors, cognitive aging researchers may better understand individual differences in cognitive trajectories. This is supported by data from a confirmatory factor analysis showing that cognitive reserve can be identified as a separate factor of individual differences beyond traditional cognitive domains such as memory, executive function, and psychomotor speed (Siedlecki et al., 2009). On the other hand, it is important to note that variables thought to explain brain reserve capacity, such as age, innate intelligence, genetic factors, head size, and brain volume—the latter two used as proxies for the number of neurons and the density of synapses—influence not only cognitive outcomes but also the very elements of cognitive reserve such as education and occupational attainment (e.g., greater intelligence influences educational and occupational attainment) (Schofield et al., 1997; Whalley et al., 2000; Reed et al., 2010). Thus, studies of cognitive aging are likely to benefit from including measures that account as fully as possible for brain and cognitive reserve (Jones et al., 2011).

A host of other socio-demographic factors have been linked to healthy cognitive and physical aging. The benefits of physical exercise, healthy nutrition and lifestyle and close interpersonal relationships are well described and have been reviewed elsewhere (Ball and Birge, 2002). An important point is that while some studies have examined the individual contribution of each factor, they are not randomly distributed in the population and tend to occur together in the same individual (Poortinga, 2007). Therefore, a careful assessment of these psychosocial and behavioral factors is an important consideration in both research subject selection and clinical diagnostic evaluation.

## Sensory function and cognition

The aging process has a significant impact on sensory functions, which then can adversely—but indirectly—affect cognition. Older adults show considerably more variability in visual and auditory function than the young (Baltes and Lindenberger, 1997). Downstream effects of sensory loss can mimic the presence or mask the severity of primary cognitive changes associated with brain aging. There is also evidence suggesting that age-related changes in sensory processing and cognition may share a common pathophysiologic basis (Baltes and Lindenberger, 1997).

Age-related changes in the optical properties of the eyes are substantial. Ocular disorders, including cataracts and glaucoma, are extremely prevalent in the aging population; they lead to subtle visual processing changes in domains like contrast sensitivity that are not routinely assessed in cognitive studies (Jackson and Owsley, 2003). However, when the visual changes of cataracts are simulated, they lead to measurable cognitive performance decrements (Wood et al., 2010). Vision in older adults fluctuates, especially in response to the amount of illumination; when visual performance is low, there are associated deficits in memory and other cognitive functions (Weatherbee et al., 2009).

As with vision, there are age-related impairments arising from the auditory sensory apparatus. Poor hearing, especially for the higher frequencies, is a nearly ubiquitous finding among older adults. Like vision, it shows accelerating decline in older age cohorts. Hearing loss is associated with lower Mini–Mental State Examination (MMSE; Folstein et al., 1975) scores and lower performance on neuropsychological tests of memory and executive function (Lin et al., 2011).

The NIH Toolbox initiative (see overview paper by Roberson et al., this volume) has recognized the importance of sensory assessments in understanding cognitive ability, but unfortunately it includes only acuity as a direct test of visual performance. This is likely to underestimate the contribution of visual impairment relative to cognition in aging (Jackson and Owsley, 2003). Experimenters can use a number of techniques beyond screening for visual and auditory deficits to ameliorate the effect of age-related sensory deficits. Good visual stimulus designs avoid low contrast or luminance, short wavelength colors (violet and blue), and high spatial or temporal frequencies (Scialfa, 2002). Researchers seeking additional information on the origins and effects of age-related sensory function on cognition may find Schneider and Pichora-Fuller's (Schneider and Pichora-Fuller, 2000) extensive review helpful.

## Effect of comorbidities

The boundaries between risk factors for cognitive decline and causes of that decline are becoming increasingly blurred. This is true for both genetic factors and illness states that predispose to the development of dementia. The ε-4 allele of Apolipoprotein E (*APOE 4*) is well recognized as a genetic risk factor for sporadic Alzheimer disease. However, middle-aged asymptomatic *APOE 4* carriers in the Framingham Offspring Cohort showed a more rapid decline in logical memory performance compared to non-carriers, without showing concomitant changes in MRI markers of brain aging (Debette et al., 2009). The following paragraphs address common comorbidities known to influence cognition and dementia risk.

### Cardiovascular factors

Modifiable cardiovascular risk factors such as hypertension, diabetes, dyslipidemia, smoking, and obesity are nearly ubiquitous among older adults in developed nations and have been associated to varying degrees with greater risk of dementia. Less is known about the effect of these factors on cognitive decline in normal aging, especially in the oldest old where hypertension appears to be the most prevalent risk factor and has been associated with worse cognitive performance among the non-demented (Peltz et al., 2012). Further, it is important to note that even without structural brain changes, cardiovascular risk factors have been associated with worse cognition. Participants with hypertension in the Framingham Offspring Cohort showed greater declines on executive function tests (Trailmaking Tests), an observation that was not altered by adjusting for markers of white matter ischemia on MRI or baseline education (Debette et al., 2011). The same study found that midlife obesity was associated with more rapid decline in Trailmaking Test performance. Thus, it is important to consider cardiovascular risk factor status when designing studies of cognitive aging.

### Mood disorders

Research examining mood symptoms among elderly non-demented individuals has shown that about one third experience low level depressive symptoms, referred to as subthreshold or subsyndromal depression (Lyness et al., 2007) However, because affected individuals may not recognize or voice such mental health symptoms, identifying these concernsin study samples can be quite difficult. There are several reasons why it is important to carefully assess mood state in studies of cognition in aging. First, depression, anxiety, and apathy can directly impact cognitive performance, masking a subject's true ability. For example, depression has significant effects on episodic memory and executive function (Austin et al., 2001), which are frequently domains of interest in studies of cognitive aging. Second, extreme symptoms are not always apparent on screening instruments like the Geriatric Depression Scale (Sheikh and Yesavage, 1986) or the revised Beck Depression Inventory (Beck et al., 1996). These scales are used frequently, but reliance on a cut score to identify a mood state sufficient to affect cognition has shortcomings. Individuals may endorse a severe symptom on a questionnaire but still fall below the cut score necessary for a clinical diagnosis. This is particularly true among participants with more minor depressive syndromes, for whom false negative rates on this type of scale can exceed 20% (Gallagher et al., 1983). Therefore, it may be valuable to examine the individual items on mood questionnaires as well as the total score. Third, changes in mood states may be an important marker of early or incipient cognitive decline and thereby undermine conclusions about what is “normal” cognition (Rosenberg et al., 2010).

### Drugs and alcohol

Although often not assessed in detail, older adults are especially at risk for potential complications and disorders associated with the use of prescription and over the counter drugs, including alcohol (Powell, 2004). A large proportion of older adults are on multiple medications for systemic diseases, many of which have the potential to interfere with cognition. Researchers and clinicians need to be aware of possible drug and alcohol effects when assessing cognition in the elderly. A thorough review of medications, recreational drugs, and alcohol usage will allow investigators to statistically control for potential confounders that may interfere with the cognitive abilities under study. For instance, there is an extensive literature demonstrating cognitive impairments following long-term use of antianxiety drugs, especially the benzodiazepines (Green, 2000; Powell, 2004). Similarly, a community-based study conducted in France revealed that 80% of an elderly sample who chronically used anticholinergic drugs, like those frequently used to treat urinary incontinence, showed detectable but non-progressive cognitive impairments (Ancelin et al., 2006).

## Screening techniques

The preceding sections have discussed general considerations in study design and subject selection. At a more practical level, researchers must characterize the cognitive performance of their participants as appropriate for age, or as impaired. While many studies will use detailed experimental approaches to assess specific cognitive domains, most investigations use brief measures that include performance across a range of cognitive skills to determine subject eligibility.

Dozens of standardized tests have been developed to screen for abnormalities in human cognition. However, their identified purpose has generally been either to identify, or exclude, individuals with disease-based alterations in cognition, such as dementia, rather than identify patterns of change associated with the aging process. The American Psychological Association *Practice Guideline for Evaluation of Dementia and Age-Related Cognitive Decline* addresses the limitations of standardized tests for detecting age-related changes in cognition and makes several general recommendations regarding limitations of standardized tests for detecting age-related changes in cognition (see http://www.apa.org/practice/guidelines/dementia.aspx#). The guideline suggests that cut-scores on screening tests offer reasonable sensitivity to dementia, but perform less well in classifying the earliest cognitive changes associated with “pre-clinical dementia.”

### In person testing

The MMSE, developed in the 1970 s, is the most widely employed instrument for assessing cognition in the elderly (Folstein et al., 1975). It is short, consisting of 11 questions totaling 30 points, and performance is not timed. Although originally designed for psychogeriatric patients, the MMSE is currently used as a screening instrument for patients with a wide range of neurologic and medical disorders. Its major uses are to serve as a screen for general cognitive impairment, assess the degree of impairment and prospectively monitor change. A normal MMSE provides good utility for excluding dementia in community and primary care settings, with negative predictive values in the 95–98% range (Mitchell, 2009). Unfortunately, the instrument is not sensitive for discriminating between age-related cognitive change, mild cognitive impairment and early dementia (Ismail et al., 2010).

There are clear advantages to using the MMSE, which include its straightforward administration, the use of published cut-scores as well as age and education corrected norms, and numerous international translations. However, there are also limitations, the most significant being that the MMSE is frequently used as a diagnostic measure and not, as originally intended, as a screening instrument. Other limitations of the MMSE include its heavy reliance on verbal abilities, (penalizing those with language problems and limited education) and a lack of agreement with regard to the best cut score.

Other screening tools have been developed that address some of the MMSE limitations in identifying milder cognitive impairments. These include the Modified MMSE (3MS; Teng and Chui, 1987), Montreal Cognitive Assessment (MoCA; Nasreddine et al., 2005), the St. Louis University Mental Status Test (SLUMS; Tariq et al., 2006). The 3MS, an adaptation of the MMSE, adds four items and uses an expanded scoring system (0–100). The MoCA was specifically developed as a screen for mild cognitive impairment in the general population and has been shown to have excellent test–retest reliability, inter-rater reliability, and convergent validity. Another strength is that the MoCA measures a broader range of cognitive abilities than the MMSE, including executive function. Like the MMSE, the SLUMS is built around 11 items and a 30-point scoring system. However, it showed greater sensitivity to milder cognitive impairments in people not meeting dementia criteria. Although each has been compared to the MMSE, further head-to-head testing among these alternatives has not been systematically studied. Additional instruments in less common use for studies of cognitive aging were recently reviewed by Ismail and colleagues (2010). Based on their intended role to identify illness (rather than characterize age-related change) and the many variables that influence performance within age cohorts noted above, there appears to be an insufficient data to make an evidence-based recommendation for a specific screening instrument to establish normal cognition across aging cohorts.

Mental status screening instruments are not meant to take the place of a more thorough diagnostic work up in clinical settings. A carefully selected neuropsychological test battery will improve diagnostic sensitivity and specificity while providing a comprehensive evaluation of mental status function. Guidelines for selecting individual cognitive domains and relevant assessment examples that could be incorporated into a comprehensive test battery were recently published by the DSM-V Neurocognitive Work Group (Ganguli et al., 2011). The DSM-V, to be published in 2013, will use the new diagnostic category *Neurocognitive Disorders*, to replace the older category of “Delirium, Dementia and Amnestic, and Other Geriatric Disorders.” This newer diagnostic entity will be further subdivided into three syndromes: Delirium, Major Cognitive Disorder, and Minor Cognitive Disorder. The six principal cognitive domains that are recommended for documenting and quantifying the degree of impairment include complex attention, executive ability, learning and memory, expressive and receptive language, visuoconstructional-perceptual ability and social cognition.

### Telephone screening

Some investigators studying variables relevant to cognitive aging need to classify the cognitive state of participants without in-person assessments. The Telephone Interview of Cognitive Status-Modified (TICS-m) is a mental state assessment designed to screen for cognitive impairment by telephone. The TICS-m showed modest correlations with a comprehensive neuropsychological assessment in older persons without dementia (Van Den Berg et al., 2012). However, its utility for identifying subtle decrements in individuals is limited and it may misclassify as many as 40% of participants, typically in the direction of assigning dementia diagnosis to mild deficits (Cherbuin et al., 2008). This may be especially problematic for researchers studying the subtle changes associated with cognitive aging. The Memory Impairment Scale (MIS; Buschke et al., 1999) is a neuropsychological screening test that was adapted for telephone administration, retaining good psychometric properties. In a sample of 300 participants (mean age 80) with 9% dementia prevalence the MIS showed sensitivity of 78%, specificity of 93% and a misclassification rate of 8% (Lipton et al., 2003).

### Self-rated and informant-reported scales

Subjective memory (or cognitive) complaints (SMC) are common in older adults, being reported by up to 88% of individuals older than 85 years (Larrabee and Crook, 1994). This is important because SMC have been associated with a greater risk of dementia in some studies, while data is limited regarding age-related cognitive decline (Waldorff et al., 2012). Thus, SMC do not necessarily reflect meaningful variations from normal performance for age. Cherbuin and colleagues (2008) conducted a systematic review of available self- and informant-report instruments to assess cognitive abilities. Only two instruments, the Concord Informant Dementia Scale (CIDS; Waite et al., 1998) and the Informant Questionnaire on Cognitive Decline in the Elderly (IQCODE; Jorm, 2004) met all selection criteria for their analysis. The CIDS and IQCODE as well as the MIS (see above) had a misclassification rate and negative predictive value better than the MMSE in samples with dementia prevalence around 10%; additionally, the sensitivity and specificity of the three instruments were similar to those of the MMSE (Cherbuin et al., 2008).

The CIDS is a 31-item instrument that assesses changes in cognition over a 5 year reference period. A 12-item short form (SCIDS; Waite et al., 1998) is also available. Both CIDS and SCIDS scores discriminate between demented and non-demented people in a community sample and correlate with neuropsychological test performance; furthermore, they appear to be less influenced by educational level than MMSE (Waite et al., 1998).

The IQCODE is a more widely known questionnaire with numerous translations to other languages. The instrument originally consisted of 26 questions; a shorter 16-question form performs equivalently to the long form and is generally preferred (Jorm, 2004). The questions are intended to be answered by an informant who has known the subject for more than ten years. They address short-term and long-term memory, orientation in time and space, financial awareness, learning, and executive skills. Education, pre-morbid ability and language proficiency have little impact on IQCODE scores (Jorm and Jacomb, 1989) but a participant's anxiety or depression may influence the assessment (Jorm, 2004).

One instrument not included in Cherbuin and colleagues (2008) review is the AD8 questionnaire, which is a simple 8-question, informant-based, survey of memory, orientation, judgment, and function. Among a sample with a 0.38 prevalence of very mild dementia, endorsement of two or more AD8 items provided sensitivity of 74%, and specificity of 86% for discriminating between normal and impaired (Galvin et al., 2005). A self-rated form of the AD8 discriminates individuals with dementia from those without, especially for mild dementia; self-rated AD8 was somewhat less effective at this differentiation than the informant reported method (Galvin et al., 2007).

### Computerized testing

Technology assisted assessments (e.g., computer administered cognitive batteries, tele-health visits) are rapidly advancing but appropriate psychometric properties and normative data are nascent. These technologies may have significant advantages for older persons with limited mobility or health-care access, but may also disadvantage older persons with limited experience and expertise interacting with technology. There are distinct advantages to using computerized assessment in research studies. Test stimuli can be presented in a highly rigorous and reproducible fashion, while both response patterns and response latency patterns can be systematically recorded and analyzed. From the data-management perspective, data are automatically tabulated and stored for future use. In addition, large numbers of participants can be easily screened without introducing human factors concerns such as variability across multiple practitioners, examiner bias, and inter-rater reliability.

However, there are also disadvantages associated with computer-based assessments. Some computerized tests have not been validated and do not meet established psychometric testing standards. Participants with poor motor control, slowed reaction time, or sensory deficits may be unnecessarily penalized when attempting to solve computer generated tasks Individuals who are not putting forth their best effort, lack motivation, or attempting to feign cannot be easily differentiated from those who have genuine cognitive impairments. Another consideration is that older adults, many of whom have little or no experience using technology, are typically less comfortable with computerized testing (Broglio et al., 2007). Although this latter issue will most likely abate over the next decades, there will continue to be wide cultural and socio-economic differences in familiarity with computers which, in turn, may limit the generalizability and reproducibility of findings from this method.

A major limitation of scores based on automated tabulation of the subject's responses is that the investigator/clinician knows only whether the item was successfully passed or failed, not how the problem was solved. Knowledge of the participant's approach can be critically important when the investigator is trying to understand the reasons underlying task failure and the emphasis is on obtaining information regarding different cognitive profiles or identifying patterns of cognitive deficits (Lezak, 1995). Although computerized tests are supposed to measure the same abilities as pencil and paper tasks, they do not necessarily provide comparable information. Furthermore, contrary to expectation, computerized assessments do not necessarily result in high test–retest reliability. Schatz and Browndyke (2002) suggested that the automated presentation of stimuli, combined with limited interaction between the examiner and examinee provides such limited resemblance to traditional assessment methods that the two forms of testing will never be truly equivalent.

## Conclusions

Human subjects research on cognitive aging is challenging. Controlling for the many confounding factors increases the difficulty of recruitment, the complexity of data collection and analysis, and burden on participants, investigators, or both. Stricter subject selection criteria for confounding factors such as demographics or concomitant disease may simplify the approach to a specific research question about cognition in aging while simultaneously reducing the applicability of its results to end users like practicing physicians and their patients with memory concerns. Nonetheless, an investigator who understands the complicating factors, as well as the strengths and weaknesses of specific assessment methods, will be better positioned to generate the most reproducible and meaningful results.

### Conflict of interest statement

The authors declare that the research was conducted in the absence of any commercial or financial relationships that could be construed as a potential conflict of interest.
